# Long-Term Fipronil Treatment Induces Hyperactivity in Female Mice

**DOI:** 10.3390/ijerph17051579

**Published:** 2020-02-29

**Authors:** Svenja Koslowski, Camille Latapy, Pierrïck Auvray, Marc Blondel, Laurent Meijer

**Affiliations:** 1 Perha Pharmaceuticals & ManRos Therapeutics, Centre de Perharidy, 29680 Roscoff, Bretagne, France; svenja.koslowski@gmail.com; 2 C.RIS Pharma, Parc Technopolitain, Atalante Saint Malo, 35400 Saint Malo, France; c.latapy@c-rispharma.com (C.L.); p.auvray@c-rispharma.com (P.A.); 3 Université Brest, Inserm, EFS, UMR1078, GGB, F-29200 Brest, France; marc.blondel@univ-brest.fr; 4 Service de Génétique clinique et de Biologie de la reproduction, CHRU (Centre Hospitalier Régional et Universitaire), F-29200 Brest, France

**Keywords:** fipronil, hyperlocomotion, memory impairment, dementia, insecticide

## Abstract

Fipronil is an insecticide widely used for veterinary and agricultural purposes. While its insecticidal properties mostly rely on its high affinity antagonistic activity on insect γ aminobutyric acid (GABA) receptors, fipronil and its main metabolite fipronil sulfone nevertheless display non-negligible affinity for mammalian GABA_A_ receptor. As several environmental toxicants have been shown to raise the risk of developing various neurodegenerative disorders, the aim of this study was to evaluate whether long-term low dose administration of fipronil could lead to cognitive deficiencies. Our results indicate that long-term fipronil treatment leads to behavioral perturbations in mice, indicating an accumulative effect of sustained exposure to low dose of fipronil. Although no memory impairment was observed during the course of our study, we noticed a significant hyperlocomotion behavior after 43 weeks of weekly fipronil administration, which is consistent with its direct effect on the GABAergic system.

## 1. Introduction

Dementia is an increasing problem in Western countries, given that aging is a major risk factor for developing this type of impairment [1,2]. Hence, as human life expectancy increases, dementia develops as a major problem. In 2018, over 50 million people lived with dementia worldwide and in 2015 it was estimated that more than 9.9 million new cases arise every year [3,4]. In addition to representing a major issue for public health, dementia also has a profound financial impact, as worldwide societal economic costs related to care for dementia patients were estimated to USD 818 billion in 2015 [4]. Aged-related dementia can most commonly be related to neurodegenerative diseases, the most frequent one being Alzheimer’s disease (AD). Recent studies carried out on dementia patients in Sweden and North America revealed that Alzheimer’s disease alone accounted for > 40% of the dementia cases studied [5,6]. 

As for AD, the exact underlying causes for the development of neurodegenerative diseases remain unknown in the majority of cases (for review see [7]). However, growing bodies of evidence suggest that chronic exposure to certain chemical compounds, notably pesticides and some metals, can be neurotoxic and favor the development of neurodegenerative disorders (see [8,9,10] and references therein). Taking into account that humans are susceptible to be exposed, even at low doses, to plant and animal protection products or their transformation products in various ways (e.g., nutrition, contact with the animals in the case of pets, proximity to agricultural holdings) and that some of those substances or their metabolites are prone to bioaccumulation, these findings represent a serious concern when one considers a potential lifelong exposition to those compounds. During the evaluation of active substances for clinical testing authorization, critical endpoints for human health are assessed based on in vitro and in vivo studies carried out on animals. While European directives recommend the evaluation of some behavioral criteria (e.g., motor activity) in order to estimate the risk of neurotoxicity in the case of human pharmaceuticals [11,12], this is not the case for plant protection products, to which humans are most often exposed unconsciously [13,14]. In addition, the evaluation of the putative effects of a given compound on cognitive functions, like learning and memory, is not considered to be crucial, though some authors have advocated for its inclusion in the core battery of behavioral tests for pharmacological products [15,16]. 

Fipronil is a widely used phenylpyrazol insecticide employed for agricultural and veterinary purposes. It came to broad public attention when, in 2017, despite its prohibited use in food-producing animals, fipronil-contaminated eggs were found in several European countries [17]. 

Fipronil, and its main metabolite fipronil sulfone, act as inhibitors of insect GABA receptors [18,19,20]. By preventing the binding of GABA (γ-aminobutyric acid) to its receptor, the compound blocks the inhibitory function of GABA in the central nervous system (CNS) and thus leads, at low doses, to neuronal hyperexcitation and, at high doses, to the paralysis and death of insects. The selectivity of fipronil is due to its notably higher affinity for insect GABA receptors than for mammalian GABA_A_ receptors [18,19,21,22]. Contributing to its selectivity, fipronil has been reported to be a potent inhibitor of the invertebrate specific glutamate-activated chloride channel [23,24]. After exposure, fipronil is rapidly oxidized by cytochrome P450s to form fipronil sulfone, its major metabolite, which presents a similar bioactivity than the parent compound and is prone to accumulate in adipose tissues [19,25,26,27,28]. Both fipronil and fipronil sulfone were reported to cross the blood–brain barrier (BBB) in rats and mice [19,29,30]. Importantly, a recent study showed that fipronil and other pyrazole insecticides can stimulate the production of the toxic amyloid β 1-42 peptide in vitro [31], suggesting a potential link between fipronil exposure and the triggering of Alzheimer disease, but this issue has not been solved yet. Hence, the aim of this study was to determine whether chronic low-dose exposure to fipronil could cause memory impairment in Swiss mice. This was tested by submitting vehicle- and fipronil-treated animals to the Morris water maze task, a test of spatial memory commonly used in rodent models of Alzheimer’s disease, at different treatment durations [32,33]. In order to exclude whether the observed effects are due to differences in general activity or anxiety, as hyperactivity has already been observed after chronic daily administration of fipronil in some rabbits and dogs [34], all animals were also submitted to the open-field test [35,36].

## 2. Materials and Methods

### 2.1. Ethics Statement 

Animal care and experimental practices were conducted at C.RIS Pharma in Saint Malo, France. The Institutional Animal Care and Use Committee (Comité éthique du groupe Cellis Pharma) approved the protocol (study number CP-2016160.PART II) prior to the initiation of the experimentations according to French regulation (article R214-217 to 221, code rural). 

### 2.2. Animals

In humans, women show a higher prevalence of Alzheimer’s disease than men [37]. Furthermore, in transgenic murine models of AD, female mice are more susceptible to plaques and tangles than males, displaying, therefore, an earlier disease onset [38]. For those reasons, we chose to carry out our study using female, rather than male, wild-type mice. Female Swiss (RjOrl:SWISS) mice from Janvier (Le Genest- Saint-Isle, France) were obtained at an age of 7 weeks. The animals were housed under a controlled temperature (22 ± 3 °C), humidity (50 ± 20%), photoperiod (12 h light/12 h dark) and air exchange. Treatment began after an acclimation period of 6 d. The Institutional Animal Care and Use Committee (Comité éthique du groupe Cellis Pharma) approved the protocol (study number CP-2016160.PART II) prior to the initiation of the experimentations.

### 2.3. Chemicals and Treatment Preparation 

Fipronil was purchased from Carbosynth (Compton, Berkshire, UK, # FA23290). Fipronil was dissolved at a concentration of 25 mg/mL in dimethyl sulfoxide (DMSO) (Sigma-Aldrich, Lyon, France, # 276855) and further diluted in corn oil (Sigma-Aldrich, Lyon, France, # C8267) to a final concentration of 1 mg/mL of fipronil in 4% DMSO. The vehicle consisted of corn oil, 4% DMSO. All treatment solutions were prepared extemporaneously to use. 

### 2.4. Animal Treatments and Treatment Groups

Animals were treated with fipronil (10 mg/kg) or vehicle once a week (Wednesdays) during work hours (9 a.m. – 6 p.m.). Treatment solutions were given to animals by oral administration (gavage) at 10 mL/kg. For the determination of the treatment dose and schedule, the lethal dose, 50% (LD_50_) of 91 mg/kg in mice (single oral administration, [28]) was taken into account. Further, in a preliminary study published in a previous article, we measured fipronil and fipronil sulfone in plasma, brain and adipose tissues of mice and rats following single and repeated oral administrations of fipronil at 10 mg/kg [31]. The results clearly show rapid transformation of fipronil into fipronil sulfone and long-lasting accumulation in brain and adipose tissue. The half-life of fipronil sulfone was found to be 14 ± 3, 17 ± 2, and 26 ± 3 d in the plasma, brain, and adipose tissue, respectively. The treatment schedule (once a week via oral dose) was employed in order to permit metabolization and slow the bioaccumulation of the compounds according to the half-life determined in the previous study. The oral administration route, rather than food or dermal intake of fipronil, was chosen in order to control the dose administrated. A relatively high dose of fipronil was chosen in order to avoid the daily treatment of animals (which might influence the results of behavior testing and animal well-being) and to adapt to the considerably shorter lifespan of mice as compared to humans (where fipronil and its metabolites can possibly accumulate over decades).

Treatment groups of ten mice per group were defined by the treatment duration. Vehicle- and fipronil-treated groups were sacrificed after 48 weeks of treatment. Animals were first anesthetized with isoflurane and then exsanguinated via intracardiac puncture, a cervical dislocation was realized afterwards to assure the animal’s death and an autopsy was carried out. 

### 2.5. Behavioral Testing

In order to assess the effects of fipronil treatment on basal motor activity and memory, two distinct behavior tests were carried out: the open-field test, which is classically used to monitor basal activity and anxiety parameters, and the Morris water maze task, which is generally considered to report spatial memory [39,40]. Indeed, the Morris water maze task is dependent on hippocampal function and, as such, is frequently used to assess memory in transgenic models of Alzheimer’s disease [41].

**Figure 1:**
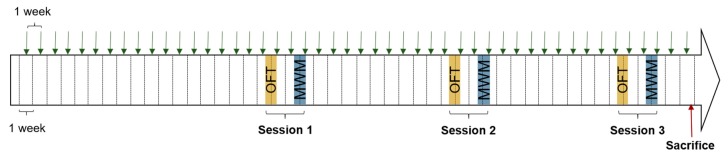
Schematic representation of the study design. Green arrows: fipronil or vehicle administration; OFT: open-field test; MWM: Morris water maze test.

For each behavior test, three sessions were carried out, each at three different timepoints during the course of the study, in order to assess the kinetics of behavioral impairment potentially induced, or not, by long-term pesticide treatment (Figure 1). 

### 2.6. Open-Field Testing (OFT)

Three sessions of open-field testing were carried out during the study after the animals had received, respectively, 18, 31 and 43 weekly treatments (by vehicle or fipronil). Animals were filmed and the videos were used to assess basal activity and anxiety parameters. All sessions have been carried out by the same experimenter. In order to limit the potential interference of environmental factors on animal behavior, sessions took place at least two days after the last cage change, on days where no further manipulation of animals was scheduled. In order to limit the influence of animal manipulation on animal behavior and observe the effects of prolonged treatment rather than those of the acute dose administered, no behavior testing was carried out on the day of treatment; corn oil-treated animals were tested on the day following the last administration, fipronil-treated animals were tested on the fifth day following the administration.

The open-field testing was carried out in an open-field arena of dark grey color measuring 40 cm in length, depth and height. 

Animals were placed in the behavior testing room at least 30 min before the start of the test. They were then placed for 30 min in the empty open-field arena which they could explore freely. The open-field arena was cleaned with Axis Hygie-Net 5% solution (Laboratoires A.C.I., Cabries, France) between each individual. 

### 2.7. Morris Water Maze (MWM)

Three sessions of the Morris water maze task were carried out during the study after the animals had received, respectively, 20, 33 and 45 weekly treatments (by vehicle or fipronil). Animals were filmed and the videos were used to assess basal activity and memory parameters. All sessions have been carried out by the same experimenter and started the day following the last treatment administration, no treatment was administered during the seven days of testing. In order to limit the learning effect by repeated testing performed on the same groups of animals, platform and starting position sequences, as well as visual cue positions, were changed for each session (Appendix A Table A1). 

The Morris water maze task for assessment of spatial learning was carried out in a circular pool of 130 cm in diameter and 50 cm in height. The target platform was 15 cm in diameter, submerged 1-1.5 cm under the water surface and located 25 ± 2 cm from the wall of the pool. Green non-toxic tempera paint (Lefranc and Bourgeois, Redimix Tempera Paint, Le Mans, France) was added to the water to make it opaque. Water temperature during the test stayed constant at 20 ± 2 °C.

Starting positions for animals were marked on the outside of the pool as north (N), north-east (NE), east (E), south-east (SE), south (S), south-west (SW), west (W) and north-west (NW). The whole apparatus was surrounded by a green tarp fixed at the ceiling in order to prohibit any influence of any unintentional spatial room cues. The visual cues for MWM spatial memory task where placed directly on the tarp. 

Our protocol consisted of 7 d of continuous testing, where the first day of the test served for the habituation of animals to the apparatus and the assessment of swimming capabilities. The second to sixth day of testing consisted of the acquisition phase, whereas on the seventh day of the MWM task a probe trial was performed. The MWM protocol started the day following fipronil- or vehicle administration and on the last day of MWM testing, after animals had carried out the probe trial, treatment was continued. Animals were placed at least 30 min before the beginning of the test in the testing room. Having finished their trials of the day, animals were dried with paper towels and then placed for at least 10 min under a heat lamp to avoid hypothermia. 

#### 2.7.1. Habituation

On the first day of the MWM test, no visual cues were present in the maze environment. Before the beginning of the test, all animals were placed for 15 s on the submerged platform. Then, the platform was rendered visible by a visual marker (yellow plastic cone) and each animal had five successive trials of 60 s to escape the water by mounting onto the platform. The platform and starting position changed at each trial. When the animal did not find the platform during a period of 60 s, it was placed on it. After each trial, animals were allowed to remain on the platform for 15 s before being placed at the next starting position in the tank. 

#### 2.7.2. Acquisition Phase

The acquisition phase took place from day two to six. Before the beginning of the test, visual cues were placed on the tarp surrounding the pool and the platform was positioned at the location where it would stay during the whole acquisition phase. Each animal then had five successive trials of 60 s to escape the water onto the platform, whereas the animal’s starting position changed at each trial. When the animal did not find the platform during a period of 60 s, it was placed on it. After each trial, animals were allowed to remain on the platform for 15 s before being placed at the next starting position in the tank. Starting positions were pseudo-randomized for the different days of the acquisition phase (Table A1). 

#### 2.7.3. Probe Trial

On the last day of the Morris water maze task, the platform was removed from the water, and the visual cues stayed in place. Each animal was placed in the water at the starting position opposite to the former platform location and was allowed to swim freely for 60 s. 

### 2.8. Analysis of Behavior Tasks

The videos of the behavior testing sessions were analyzed with ANYMAZE software (Stoelting Co., Dublin, Ireland).

For the open-field task, the total distance travelled, mean speed, time mobile and time spent at distinct locations of the open-field arena (center, border, corner) were determined. 

For the MWM task, the acquisition phase and probe trial were analyzed. During the acquisition phase, mean speed and mean distance travelled until the end of the trial (attained either by entering the island zone or after 60 s have passed) were analyzed. During the probe trial, the mean speed, relative amount of distance travelled in the target quadrant (former platform location) and number of entries to the former platform location were analyzed. For all behavior tasks analyzed, *n* = 10 for the vehicle-treated group of animals, and *n* = 9 for fipronil-treated animals.

### 2.9. Statistical Analysis

The results are expressed as mean ± SD. Statistical analysis was carried out using GraphPad Prism version 6 for Windows (GraphPad Software, La Jolla California, CA, USA). Two-way repeated measures (RM) ANOVA, with the two factors being treatment type and treatment duration, were applied for OFT mobility (total distance travelled, relative time spent mobile), thigmotaxis (per position), MWM general activity and speed, as well as MWM target platform entries during the probe trial. This test was used to reveal whether treatment type or study duration had a significant influence on the results. Tukey’s multiple comparisons post-hoc test was applied in order to compare intragroup variations between sessions. Two-way RM ANOVA was also applied to analyze the learning in the MWM training phase for each session, with the factors being treatment type and testing day. Tukey’s multiple comparisons post-hoc test was applied to analyze whether the animals’ performance differed significantly between the days of training. Where subject matching was not significant (*p* > 0.05), as reported by two-way RM ANOVA, a two-way ANOVA without considering repeated measures was conducted and, as before, Tukey’s multiple comparisons post-hoc test was applied. This was the case for the analysis of mean speed in the OFT and the relative time spent and distance travelled in the target quadrant during the probe trial of the MWM. For each session of behavior testing, the performance of both treatment groups in the different parameters measured was compared via Student’s t-test. Further, in order to analyze the relative time spent and distance travelled in the target quadrant during the probe trial, a Student’s t-test against a hypothetical value was used. The performance of each group at each session was compared against the hypothetical value of 25. This value derives from the supposition that, if no training to find the quadrant had occurred, the animals of each group would, on average, explore equally each quadrant of the pool. Thus, if no learning of the platform position had occurred, animals would spend about 25% of the trial time, or travel about 25% of the total distance travelled during this trial, in each quadrant of the pool, not distinguishing the former platform quadrant. Only significant differences are reported in the text, when no p-value is mentioned the results of the statistical analysis are not significant. A *p*-value ≤ 0.05 was considered significant, with significance levels noted as: *: *p* ≤ 0.05; **: *p* ≤ 0.01; ***: *p* ≤ 0.001; ****: *p* ≤ 0.0001.

## 3. Results

### 3.1. Toxicity Associated with Fipronil Treatment

One of the fipronil-treated mice had to be sacrificed for ethical reasons in the 18th week of the study. The macroscopic examination carried out after sacrifice revealed splenomegaly, swollen lymph nodes and anemia. As it was the only animal (among a total of 10 animals treated) showing such signs during the study, we concluded that the pathology development was probably unrelated to fipronil treatment. 

No signs of acute toxicity were observed in both treatment groups during the study. In addition, there was no significant difference in weight evolution between groups during the study nor significant difference in brain weight between vehicle- and fipronil-treated animals (data not shown). Macroscopic examinations carried out after sacrifice revealed the presence of fluid filled vesicles around one or both ovaries in some vehicle- (*n* = 4) and fipronil- (*n* = 2) treated animals. No further abnormalities were found in any of the treatment groups.

### 3.2. Open-Field Testing 

#### 3.2.1. Activity of Mice

**Figure 2:**
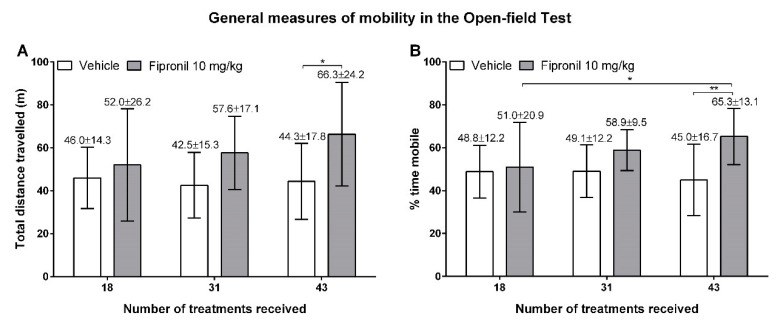
Measures of open-field activity during the 30 min of free exploration during the three sessions of open-field testing. Mean values ± SD are indicated above the corresponding bars. (**A**) Total distance (in meters) travelled by the animals (Y axis) as a function of the number of treatments (X axis). Treatment type had a modest but significant influence on the total distance travelled. (**B**) Relative mobility time of animals during the 30 min of free exploration in the percentage of the total trial duration (Y axis), as a function of the number of treatments (X axis). Treatment type had a significant effect on the relative mobility time. *: *p* ≤ 0.05; **: *p* ≤ 0.01.

The open-field test was used to assess general activity and thigmotaxis in vehicle- and fipronil-treated animals in order to reveal whether differences in those parameters could influence the further behavioral test. Fipronil treatment had a significant effect on total distance travelled during the 30 minutes of free exploration in the open-field arena (*p* = 0.0411, two-way RM ANOVA). For vehicle-treated animals, the mean total distance travelled remained constant during the three sessions of behavior testing at, respectively, 45.97 ± 14.28, 42.55 ± 15.31, and 44.32 ± 17.75 m (Figure 2A). In contrast, we could observe a clear tendency, although not statistically significant, for a treatment duration-dependent increase in the mean total distance travelled in fipronil-treated animals (respectively, 52.02 ± 26.17, 57.60 ± 17.07, 66.31 ± 24.19 m). After 43 weeks of treatment, the mean total distance travelled by animals from the fipronil-treated group was significantly higher than the one of vehicle-treated animals (*p* = 0.0361, Student t-test).

These results are consistent with our measures of mobility time, even though the effect of fipronil treatment on relative mobility time is not significant (two-way RM ANOVA). While vehicle-treated animals spent only about half of their time in the open-field arena mobile (48.81% ± 12.22%, 49.07% ± 12.23%, 44.94% ± 16.66% respectively), the relative mobility time of fipronil-treated animals increased with treatment duration (Figure 2B). Thus, fipronil-treated animals spent significantly more time mobile during the last session of open-field testing than during the first session of open-field testing (50.98% ± 20.88% vs. 65.267% ± 13.08%; *p* = 0.0297, Tukey’s multiple comparisons test). In addition, the fipronil-treated animals spent significantly more time mobile during the third and last session of open-field testing than the vehicle-treated animals (*p* = 0.0093, Student t-test). 

#### 3.2.2. Velocity Measurements

**Figure 3:**
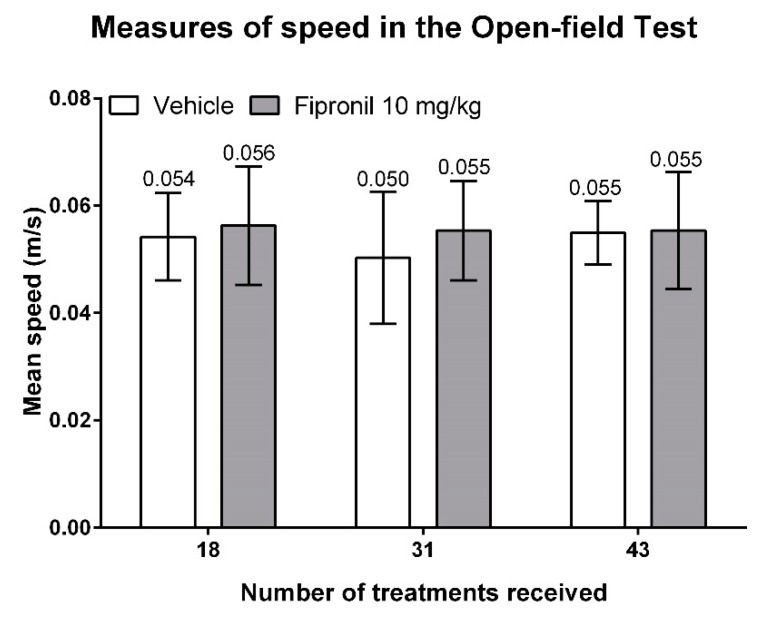
Mean speed during the 30 min of free exploration during the three sessions of open-field testing as a function of the number of treatments. Mean speed displayed by the animals in meters travelled per second (Y axis) as a function of the number of treatments (X axis). Mean values are indicated above the corresponding bars. Neither treatment type nor treatment duration had a significant effect on the mean speed of mice.

In order to evaluate whether the differences in total distance travelled observed in the open-field test were due to differences in velocity between treatment groups, we analyzed the mean speed displayed by the two treatment groups during the 30 min of free exploration in the open-field arena. Fipronil treatment had no significant effect on mean speed displayed by the animals during the 30 min of free exploration in the open-field arena, thus there was no significant difference in mean speed between treatment groups at any timepoint (two-way ANOVA, Student t-test). Hence, for both treatment groups, the mean speed remained essentially constant among the three sessions of open-field testing (vehicle-treated animals: 0.054 ± 0.008, 0.050 ± 0.012, 0.055 ± 0.006 m/s, respectively; fipronil-treated animals: 0.056 ± 0.011, 0.055 ± 0.009, 0.055 ± 0.011 m/s, respectively) (Figure 3). 

#### 3.2.3. Thigmotaxis

**Figure 4:**
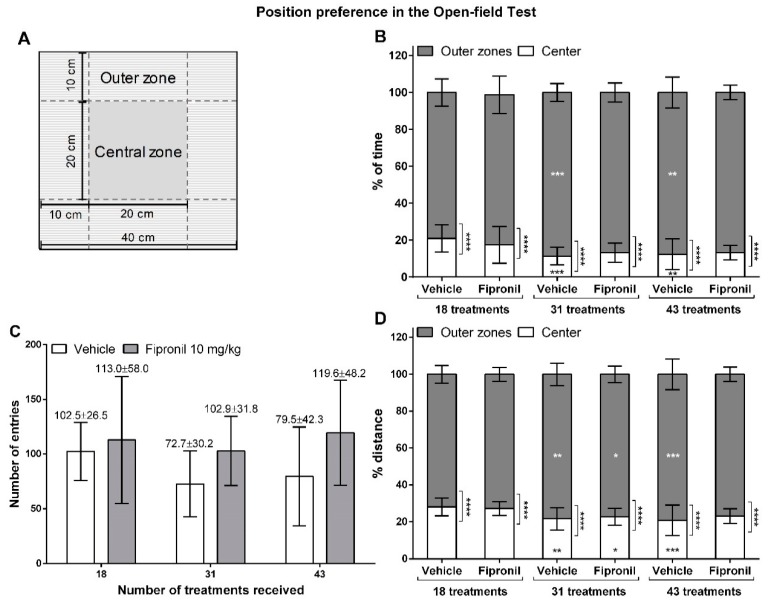
Position preference in the open-field test. (**A**) Schematic representation of the open-field arena and of the defined zones. (**B**) Relative time spent by the animals from the two treatment groups in the center and outer zones of the open-field arena during the three testing sessions. Treatment type has no significant influence on the relative time spent in the outer or central zones of the open field. (**C**) Number of entries in the central zone by the animals of the two treatment groups during the three testing sessions, mean values ± SD are indicated above the bars. Neither treatment type nor treatment duration had a significant effect on the number of entries to the central zone (and there were no significant differences between treatment groups). (**D**) Relative distance travelled by the animals from the two treatment groups in the center and outer zones of the open-field arena during the three testing sessions. Treatment type has no significant influence on the relative distance travelled in the outer or central zones of the open-field arena). *: *p* ≤ 0.05; **: *p* ≤ 0.01; ***: *p* ≤ 0.001; ****: *p* ≤ 0.0001.

In order to determine thigmotaxis of the animals, the open-field arena was virtually divided into different zones in the ANYMAZE software (Figure 4A). Outer zones were defined as all areas within 10 cm of distance from the border of the open-field arena, while the center zone was defined as a square surface of 20 cm × 20 cm in the center of the open-field arena. 

As total distance travelled and mobility time varied between treatment groups, we calculated the relative time spent and the relative distance travelled in the different zones of the open-field arena. All animals displayed a position preference in the open-field arena (*p* < 0.0001, two-way ANOVA) (Figure 4B,D, Table 1). There was no significant difference in thigmotaxis between groups for any of the open-field testing sessions (Student t-test). During all sessions, all the animals spent significantly less time and travelled significantly less distance in the center zone of the open-field arena than in the peripheric regions (Student t-test, *p* < 0.0001 for all). Both, the vehicle-treated and fipronil-treated animals, travelled significantly less distance in the center zone of the open-field arena during the second session of behavior testing (*p* = 0.0017 and *p* = 0.0426 respectively, Tukey’s multiple comparisons test). Further, the vehicle-treated animals also travelled significantly less distance in the center zone during the third session of testing than during the first (*p* = 0.0005). Considering the relative time spent in the different zones of the open-field arena, this shift is less visible: only the vehicle-treated group showed a shift of thigmotaxis during the second and third session of OFT. In both, the second and the third session, vehicle-treated animals spent significantly less time in the center zone and thus significantly more time in the outer zone of the open-field arena than during the first session (*p* = 0.0004 and *p* = 0.0014 respectively). The analysis of the number of entries into the central zone (Figure 4C) revealed that neither treatment type nor treatment duration had a significant effect on this parameter (two-way RM ANOVA) and no significant differences between the treatment groups could be revealed (Student’s t-test). However, we can see a tendency to enter the central zone less frequently with increasing treatment duration for the vehicle-treated animals, which seems coherent with the results observed for the relative time spent and distance travelled in this zone during sessions. In contrast, the number of entries to the center zone seems rather constant in the fipronil-treated groups, even though fipronil-treated animals also showed a tendency to spend less relative time and travel less relative distance in the central zone with increasing treatment duration. 

### 3.3. Morris Water Maze Task

#### 3.3.1. Acquisition of the Platform Position

The mean distance travelled during the five daily trials, rather than the time taken to find the platform, was analyzed during the learning period. Indeed, one may consider that the distance travelled before reaching the platform is less dependent on interindividual differences in swimming speed and thus represents a more accurate measure of learning. 

**Figure 5:**
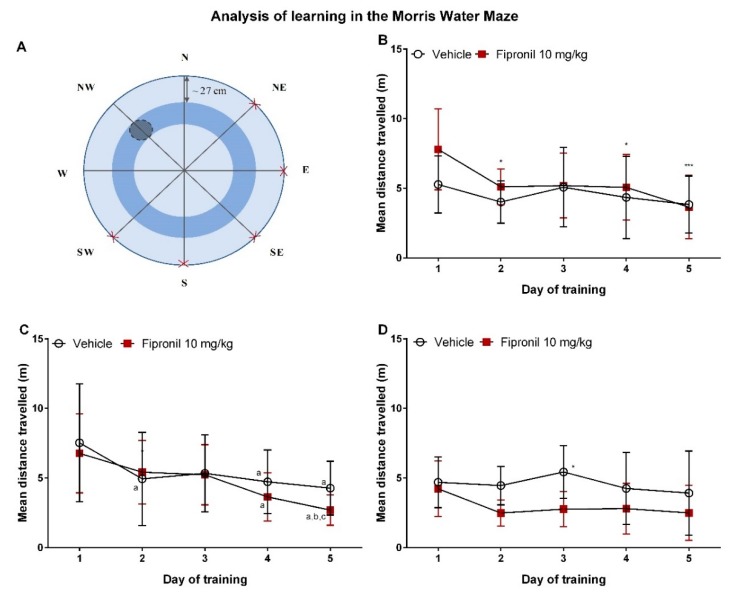
Analysis of learning in the Morris water maze. (**A**) Schematic representation of the Morris water maze tank with one possible platform position (grey circle surrounded by a dashed line) and the according possible starting positions indicated (red crosses). (**B**) Learning curve of animals during the training in the first session (performed after 20 treatments) of the Morris water maze task, as indicated by mean distance travelled in meters until island zone entry (Y axis) as a function of the day of the training (X axis). The day of training had a significant influence on the mean distance travelled (*p* = 0.0016). (**C**) Learning curve of animals during the training in the second session (performed after 33 treatments) of the Morris water maze task, as indicated by mean distance travelled in meters until island entry (Y axis) as a function of the day of the training (X axis). The day of training had a significant influence on the mean distance travelled (*p* < 0.0001; a: significantly different from day 1, b: significantly different from day 2, c: significantly different from day 3, intragroup analysis, Tukey’s multiple comparisons test) (**D**) Learning curve of animals during the training in the third session (performed after 45 treatments) of the Morris water maze task, as indicated by mean distance travelled in meters until island zone entry (Y axis) as a function of the day of the training (X-Axis). The day of training had no significant effect on the mean distance travelled whereas treatment type did (*p* = 0.0137). *: *p* ≤ 0.05; ***: *p* ≤ 0.001.

During the first two sessions of MWM testing (Figure 5B,C), there was a significant effect of the day of training but not of the treatment received by the animals on the mean distance travelled before reaching the platform (*p* = 0.0016 and *p* < 0.0001 respectively, RM two-way ANOVA). In contrast, statistical analysis indicated that during the third session (Figure 5D), there was no significant effect of the day of testing but rather of the treatment received by the animals on the mean distance travelled until island entry (*p* = 0.0137). 

During the second session, a significant decrease in mean distance travelled before island entry between the first days of training and the last days of training could be observed in both treatment groups (Tukey’s multiple comparisons test). Indeed, for the vehicle-treated group, the mean distance travelled on days 2, 4 and 5 of training was significantly lower than the mean distance travelled on day 1. For the fipronil-treated group, the mean distance travelled on days 4 and 5 of training was significantly shorter than the mean distance travelled on day 1. Furthermore, the mean distance travelled on day 5 of training was significantly lower than the mean distance travelled on days 2 and 3 of training. 

During the third session, no significant difference in the mean distance travelled before island entry could be observed between the different days of training for either group. 

#### 3.3.2. Probe Trial

**Figure 6:**
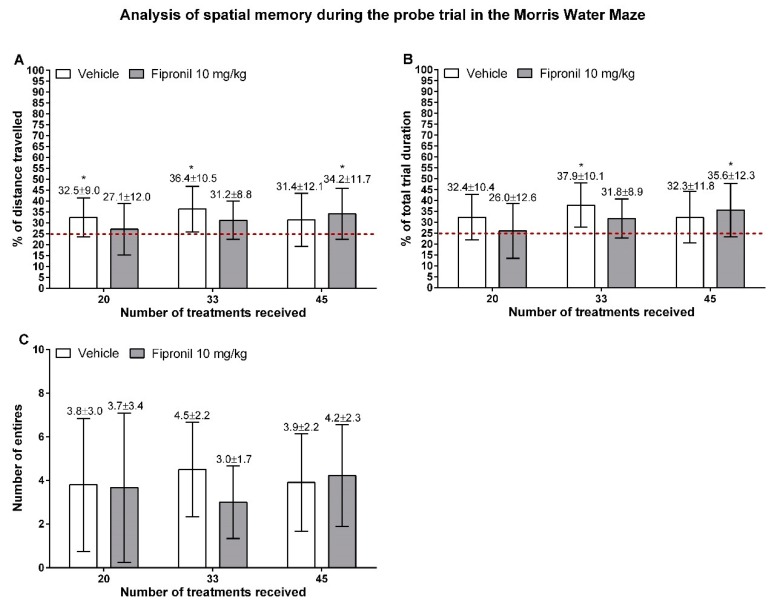
Main indicators of spatial memory as measured during the probe trial during the different sessions of the Morris water maze task. Mean values ± SD are indicated above the bars. (**A**) Relative distance travelled during the probe trial in the target quadrant (former platform location) indicated in percentage of the total distance travelled (Y axis) in function of the number of treatments (X axis). The hypothetical value of 25% travelled in the target quadrant (no recall of the former platform position) is indicated by a red dashed line. (**B**) Relative time spent during the probe trial in the target quadrant (former platform location) indicated in percentage of the total trial duration (Y axis) in function of the number of treatments (X axis). The hypothetical value of 25% travelled in the target quadrant (no recall of the former platform position) is indicated by a red dashed line. (**C**) Number of entries in the former platform location during the probe trial (Y axis) in function of the number of treatments (X axis). *: *p* ≤ 0.05.

Both, the relative time spent and the relative distance travelled in the target quadrant (former platform location) during the probe trial, were evaluated, as the first can be influenced by the swimming speed of animals while the second can be influenced by animals staying near immobile in search for the platform location. Neither treatment type nor treatment duration have a significant effect on the relative distance travelled in the target quadrant during the probe trial (two-way ANOVA) (Figure 6A). Further, no significant difference in relative distance travelled in the target quadrant could be detected between the two treatment groups in any session (Student t-test). However, a Student t-test against the hypothetical value of 25% was carried out and revealed that, during the first two sessions of the Morris water maze task, only the vehicle-treated group showed a relative distance travelled in the target quadrant significantly higher than 25% (32.53% ± 8.99% *p* = 0.0265 and 36.36% ± 10.48% *p* = 0.0075, respectively). In contrast, for the last session, only the fipronil-treated animals reached a value of relative distance travelled in the target quadrant significantly higher than 25% (34.17% ± 11.74% *p* = 0.0473).

When analyzing the relative time spent in the target quadrant (Figure 6B), the results are very similar to those observed for the relative distance travelled. However, when the Student t-test against the hypothetical value of 25% was applied in this case, neither the vehicle- nor the fipronil-treated animals displayed a relative amount of time spent in the target quadrant significantly different from 25% during the first session of the Morris water maze task (32.35% ± 10.39% *p* = 0.0522 and 26.03% ± 12.57% respectively). However, similarly to the relative distance travelled, the vehicle-treated control group had a value significantly higher than 25% during the second session of the MWM task (37.87% ± 10.15% *p* = 0.0037) and the fipronil-treated group spent significantly more than 25% of its time in the target quadrant during the last session of MWM (35.62% ± 12.28% *p* = 0.0319). 

The number of entries into the former island location during the probe trial were measured (Figure 6C) and neither treatment type nor treatment duration had a significant effect on this parameter (two-way RM ANOVA). There was no significant difference in the number of entries into the island zone between treatment groups at any timepoint investigated (Student t-test).

#### 3.3.3. Activity and Velocity

**Figure 7:**
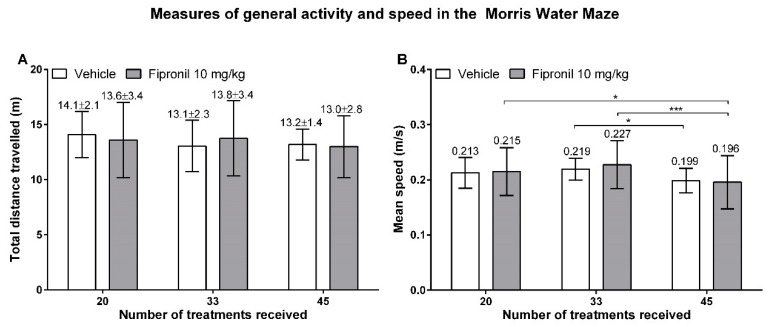
Velocity parameters measured during the Morris water maze task. (**A**) Total distance travelled during the probe trial in meters (Y axis) in function of the number of treatments (X axis). Mean values ± SD are indicated above the bars. (**B**) Mean speed as an average of the mean speed displayed during the training phase and the probe trial in meters travelled per second (Y axis) in function of the number of treatments (X axis). Mean values are indicated above the bars. The number of treatments received (corresponding to study duration) had a significant effect on the mean speed displayed by animals (Two-way RM ANOVA, *p* < 0.0001). *: *p* ≤ 0.05; ***: *p* ≤ 0.001.

The total distance travelled during the probe trial was investigated as an indicator of activity under high stress conditions (Figure 7A). The total distance travelled by the animals remained essentially stable among the three sessions of MWM (Tukey’s multiple comparisons test). The mean value of the total distance travelled during the probe trial in the three sessions of MWM testing was 13.45 ± 0.57 m for the vehicle-treated animals and 13.46 ± 0.40 m for the fipronil-treated animals, which is consistent with the fact that no significant difference in total distance travelled was found between the two treatment groups (Student t-test). 

We averaged the mean speed displayed by the animals throughout the training phase and probe trial for each session of MWM testing in order to reveal evolution of mean speed throughout the testing session or between treatment groups (Figure 7B). No significant difference in total mean speed displayed could be revealed between the vehicle-treated control animals and the fipronil-treated animals (Student t-test). Both groups displayed a significant decrease in mean speed during the third session of the MWM. Vehicle-treated animals displayed a significantly lower mean speed during the third session when compared to the second session (0.219 ± 0.020 m/s *vs.* 0.199 ± 0.022 m/s *p* = 0.0115, Tukey’s multiple comparisons test). Fipronil-treated animals displayed a significantly lower mean speed during the third session when compared to the first and second session of the MWM task (0.215 ± 0.043 m/s *vs.* 0.196 ± 0.048 m/s *p* = 0.0282; 0.227 ± 0.043 m/s *vs.* 0.196 ± 0.048 m/s *p* = 0.0002 respectively). 

## 4. Discussion

### 4.1. Toxicity of Chronic Exposure to Fipronil

At 1/9 of the LD_50_ determined for single oral administration in mice (91 mg/kg; [28]), animals chronically treated over a long period of time by fipronil showed no sign of toxicity. 

### 4.2. Fipronil Treatment Modifies General Activity in Mice

The results of this study suggest that long-term chronic administration of fipronil induces hyperactivity in animals under moderate stress conditions like in the open-field test (as compared to the MWM inducing high stress in animals due to adverse stimuli [42,43]). Compared to the control group, the fipronil-treated animals displayed significantly higher values for total distance travelled and relative time spent mobile in the OFT after 43 weeks of treatment. Further, fipronil-treated animals showed a significant increase in the time spent mobile in the OFT after 43 weeks of treatment when compared to the first session of OFT carried out after 18 weeks of treatment and a gradual increase in the relative time spent mobile is visible between OFT sessions. This gradual increase with increasing treatment duration in fipronil-treated animals is also visible for the total distance travelled in the OFT, even though only as a tendency not statistically significant. In comparison to that, the total distance travelled, and the relative time spent mobile in the OFT, remained essentially stable over time in the control group. According to these observations, we suggest that the observed effects of the fipronil treatment might potentially be due to an increasing bioaccumulation within the animals. With regard to our study setup with the treatment frequency (once a week), the treatment route (oral) and the moment of testing (on the fifth day after the last administration for the fipronil-treated animals), fipronil sulfone is most likely responsible for the observed effects. Indeed, upon oral administration, fipronil undergoes rapid metabolization in the liver to give fipronil sulfone [19,26,28], an oxidized metabolite prone to accumulation in fatty tissues and able to cross the BBB [19,29,30]. However, as in this study no quantification data for fipronil and fipronil-sulfone in the brain or fatty tissue were presented, this hypothesis needs further investigation.

Hyperactivity has already been observed after chronic daily administration of fipronil in some rabbits and dogs [34]. Results obtained by Reichel et al. in 2015 indicate that the depletion of GABAergic neurons in the hippocampus leads to hyperlocomotion in C57BL/6 mice [44]. Furthermore, decreased levels of the neurotransmitter GABA have been implicated in attention-deficit/hyperactivity disorder in children [45]. Thus, the action of fipronil may be seen as consistent with the antagonist activity of fipronil and fipronil sulfone on mammalian GABA_A_ receptors. Binding of GABA induces opening of the GABA_A_ receptor-associated Cl^-^ Channel, generally resulting in membrane hyperpolarization and thus in inhibition of action potential initiation [46]. As fipronil and fipronil sulfone binding to GABA_A_ receptors diminishes the hyperpolarizing chloride current in a dose-dependent manner they cause hyperexcitability of the neuron and high doses of fipronil have been shown to induce seizures and convulsion in animals [34]. Apart from the neurotoxic effects of fipronil and its metabolites, thyroid disruption through fipronil treatment may as well be responsible for, or contribute to, the hyperactivity observed in fipronil-treated mice in this study. It has been shown that perinatal exposure to flame retardants (polychlorinated biphenyls (PCBs) and polybrominated diphenyl ethers (PBDEs)) can cause long lasting neurodevelopmental deficits in humans and rodents—among those deficits are learning impairment and hyperactivity [47,48,49]. Those neurodevelopmental effects are thought to be induced by the disruption of thyroid hormone homeostasis—PCBs are known to induce a decrease in triiodothyronine (T_3_) and thyroxine (T_4_), while causing an increase in the thyroid stimulating hormone TSH [48,50]. Furthermore, perinatal hypothyroidism induced by propylthiouracil (PTU) administration causes long-lasting memory deficits and hyperactivity in rats [51,52]. Repeated daily fipronil administration for 14 or 28 days in adult rats and mice disrupts thyroid hormone homeostasis by significantly decreasing plasma T_3_ and T_4_ levels, while increasing plasma TSH levels and inducing cytochrome P450 (CYP) expression [53,54]. The studies of the behavioral consequences of thyroid disruption in rodents have been focused on the study of the neurodevelopmental impact (via perinatal exposure to chemicals influencing thyroid function) or chronic thyroid disfunction (e.g., via the use of transgenic mouse models) and it would be interesting to see whether adult-onset of hypothyroidism causes the same behavioral effects. In the Morris water maze test, fipronil-treated animals showed no signs of increased activity. Only the total distance travelled during the probe trial was evaluated as the total distance travelled during the other days of testing depended on learning performance. There was no significant difference in the total distance travelled between treatment groups and the total distance travelled remained essentially unchanged throughout all three sessions. Furthermore, there was no difference in mean speed displayed between the treatment groups. Variations in mean speed occurred between sessions in the same treatment group and might potentially be attributed to the effect of aging on the swimming capacity of animals. 

Together, these results suggest that the excitatory effect displayed by fipronil in the open-field test might be only relevant for non-, or moderate, stress conditions, but not for high stress conditions, as the MWM test where differences in locomotor activity and velocity between treatment groups could not be reproduced. This hypothesis is reinforced by the subjective impression of animal caretakers that fipronil-treated animals were somewhat more nervous and active than control animals during the course of the study. However, we cannot exclude the possibility that the observed hyperactivity of fipronil-treated animals in our study might be due to a lack of habituation to the novel environment. 

### 4.3. Thigmotaxis Remains Unchanged upon Fipronil Treatment

Both treatment groups spent significantly more time and travelled a significantly greater distance in the outer zones of the open-field arena than in the center zone during all sessions of behavior testing. The measure of time spent and distance travelled in the center zone of the open-field arena is an indicator of normal anxiety (as compared to anxiety disorders like social phobias) when exposed to a novel environment [35,55]. Furthermore, the number of entries into the center zone did not vary significantly between treatment groups, even though fipronil-treated animals showed a tendency to enter this zone more frequently than vehicle-treated animals during the last two sessions of testing. However, the number of entries is influenced by general activity, which differs between treatment groups, and can here only be interpreted in relation to the other parameters measured. Fipronil treatment at the dose and treatment schedule employed therefore did not modify normal anxiety in female Swiss mice. Our results rather indicate a shift in anxiety behavior over time. In our study, animals appeared to be less anxious during the first session of behavior testing than during later testing, as animals spent more time in the center zone during the first session than during the following OFT sessions. However, this effect is more visible regarding the relative distance travelled in the center zone than the relative time spent in the center zone. For vehicle-treated animals, this tendency is as well reflected in the decrease in the number of entries into the central zone between sessions, while this is not the case for fipronil-treated animals, for which this parameter stays basically constant between sessions. Hence, we think that this observation reflects the general increased activity of fipronil-treated animals, as their relative time spent and distance travelled in the open-field arena do not reveal an anxiolytic effect of fipronil-treatment as compared to vehicle-treatment. 

Shoji et al. studied the effects of aging on the evolution of numerous behavioral parameters in adult and middle-aged C57BL/6 mice (2 to 12 months of age) [56]. Their results in the open-field test indicate a non-significant tendency to spend less time in the center zone upon aging during the first 5 min of the open-field test. However, when calculated for the full duration of the test (120 min in their protocol), this tendency was inversed and older animals showed an inclination to spend more time in the center zone of the open-field arena than younger animals, which might be attributed to habituation to the novel environment. However, the overall results in their study indicate a tendency towards increased anxiety-like behavior upon aging. A similar study with a wider range of age showed that aged mice spent more time in the center of the open-field arena than young mice, starting only 10 min after the beginning of the test [57]. Shoji et al. thus suggested that, in old-aged mice, anxiety-like behavior might increase compared to young mice when exposed to a novel environment, and then decrease after a long exposure (habituation) to the same environment. The overall results of their second study again rather suggest a positive relation between age and anxiety-like behavior which is in line with our observations. Francia et al. also obtained comparable results while studying the effect of age and social status on behavior in male CD-1 mice [58].

### 4.4. Effects on Learning and Memory

During the first two sessions of MWM, significant learning of the platform position was visible during the training phase; this was not the case for the third and last session of the Morris water maze task. While, during the third session, there is no significant learning curve (decrease in the mean distance travelled until entry in the island zone), the mean distance travelled until platform entry was quite low (< 5 m travelled) on the first day of training compared to the previous two sessions of the MWM test. Thus, while a slight overall decrease in the mean distance travelled was visible between the different days of training, this decrease was not significant. In our opinion, this does not reflect a decrease in the learning capacities of animals. Indeed, when, during the first trial on the first day of training, animals found the platform position rapidly due to chance, this facilitated the finding of the platform during the following trials of the day, thus generating a lower mean distance travelled. 

As for the recall of the platform position evaluated during the probe trial, there is no significant difference in performance between the two treatment groups during any of the sessions. We evaluated both, the distance travelled and the time spent in the target quadrant (former platform location), during the probe trial as both measures have different advantages. While the distance travelled was not influenced by interindividual differences in the mean speed of animals, the latency time was not influenced by animals staying near immobile in search for the platform. However, the same tendency was visualized for both parameters: during the first two sessions the vehicle-treated group displayed a slightly better performance than the fipronil-treated group, while during the third session this tendency was reversed and the fipronil-treated animals performed slightly better than the vehicle-treated group. Furthermore, only the vehicle-treated group showed a mean distance travelled significantly higher than 25% in the target quadrant during the probe trial in the first two sessions of the MWM task, while in the third session this was observed only for the fipronil-treated group. Although the vehicle-treated animals showed a slightly better performance during the first two sessions, this tendency was reversed during the last session of testing. As there was no significant difference for any timepoint between the two treatment groups, the results do not permit us to conclude that there was any difference in learning capacities between the two treatment groups. We thus hypothesize that differences in significance of the probe trial performance as compared to the hypothetical value of 25% of distance travelled or time spent in the probe quadrant were due to chance. Of note, this hypothesis was further supported by the lack of significant difference between treatment groups in the number of former platform entries between MWM testing sessions. 

In contrast to our results, Godinho et al. showed significant memory impairment in rats after 15 d of oral treatment with fipronil at a dose of 30 mg/kg [59]. However, their pilot study included a group of male Wistar rats treated with daily oral doses of 10 mg/kg of fipronil over 15 d which did not result in significant memory impairment in those animals. Considering that in our study, mice were treated at 10 mg/kg, and only once a week, our results seem consistent with the results of their preliminary study. Even if it is difficult to compare results obtained in two different species, the fact that the acute oral LD_50_ of both rats and mice is situated around 90 mg/kg (97 mg/kg in rats vs. 91 mg/kg in mice [28]) supports this point of view. 

Furthermore, Godinho et al. used a commercial formulation of fipronil (Regent®800WG, (BASF- Agro Brazil, Sao Paulo, Brazil) and saline solution as a control. In a transgenic mouse model of Alzheimer’s disease (APP_SDL_ mouse expressing human Aβ1-40 and Aβ1-42), chronic and ad libitum administration of 1% DMSO in water has been shown to increase spatial memory in old transgenic mice through the attenuation of hippocampal neuronal hyperactivation [60]. Thus, there is a possibility that the DMSO contained in our vehicle might have attenuated memory deficits in fipronil-treated animals, even though the total dose of DMSO ingested by mice in the study published by Penazzi et al. is very likely to be higher than the dose ingested by animals in our study. At the dose administered DMSO is, however, unlikely to influence locomotor activity [61]. 

Godinho et al. also analyzed the general activity of rats, treated or not with fipronil, in the open-field test and they observed no differences in locomotor activity (evaluated as number of zones entered in 3 min of open-field testing) between fipronil treated and control animals. Another study published in 2016 showed that the dermal administration of fipronil to lactating mothers (female Wistar rats) at 1 mg/kg/day during the 7th to 14th days of lactation resulted in memory impairment in their litter [62]. Thus, the absence of memory impairment observed during our study could be due to the relatively low dose of fipronil administrated and the treatment schedule employed. 

### 4.5. Consideration of Repeated Behavior Testing

It is the general opinion that behavior testing on animals should be carried out on naive individuals in order to obtain reliable results uninfluenced by the effect of habituation (see [39] and references therein). While habituation certainly has an effect when behavior tests are carried out with only small time intervals in-between testing sessions [63,64], our results indicate no specific signs of habituation, such as less anxiety (displayed as more time spent or distance travelled in the center area of the open-field arena as an effect of habituation to the environment) during the test sessions. The general performance of the vehicle-treated animals remained nearly constant during all sessions of behavior testing and the observed differences might be caused by the effect of aging. Furthermore, there was no significant difference in crossings of the former platform position during the probe trial among sessions of MWM testing. However, a naïve group of vehicle-treated animals of the same age included in each session of behavior testing would have been necessary to prove beyond doubt that no habituation to the tests had taken place. 

Repeated elevated plus maze testing in rats has shown that while there was a habituation effect, this did not influence measures like total distance travelled. The authors of the study concluded that as long as an untreated control group is integrated in a study, repeated testing would still permit them to observe specific effects induced by the treatment with a test substance [65]. Furthermore, in our study time intervals between behavior testing sessions were nearly 3 months (11 and 12 weeks) and we changed platform position, starting positions and spatial cue arrangement between MWM testing sessions. 

When comparing the outcomes of behavior tests from different studies, one should always bear in mind that results are influenced by the strain and the sex of the animals used, as well as different environmental factors [66,67].

### 4.6. General Considerations

It is known that the GABAergic system is implicated in learning and memory acquisition (see [68] and references therein). While previous studies mostly indicated that GABA_A_ agonists, such as muscimol and diazepam, impair memory function, there is increasing evidence that inhibition of the GABAergic system could induce memory impairment as well (see [68] and references therein; [44,59,69,70]). This indicates that a balance between inhibitory and excitatory neuronal circuits is necessary for memory formation and that an imbalance in either direction could lead to impairment of cognitive functions. As shown by the results of Reichel et al., depletion of GABAergic neurons in the hippocampus leads to hyperlocomotion and abolishment of spatial learning capacities in mice [44]. Rats showed diminished learning abilities following more severe fipronil treatment than the one applied in the present study (30 mg/kg and daily administration for 15 d) [59]. In the present study, at much lower doses (10 mg/kg) and larger treatment intervals (weekly oral administration), we observed time-dependent hyperlocomotion. Hence, our results indicate that there is an accumulative effect of long-term treatment, as shown by the increase in locomotor activity in function of the treatment duration. Furthermore, this effect seems to be coherent with a direct effect on the GABAergic system, notably in the hippocampus. Importantly, with respect to the bioaccumulation potential of fipronil sulfone, we cannot exclude the possibility that an effect on the cognitive functions may have been observed with a longer treatment duration. 

We are, however, aware of the shortcomings of our study concerning the measurement of activity parameters, as the testing for differences in general activity and anxiety-like behavior among treatment groups was integrated mostly to permit reliable analysis of the MWM memory task. In order to validate our results, further studies should be carried out concerning the effect of fipronil treatment on general activity in mice. To assess whether the observed hyperactivity of fipronil-treated animals in the OFT is due to a deficit in habituation to the novel environment, an analysis of locomotor activity over time and a comparison with home cage activity should be carried out. In addition, a larger test battery including the assessment of physical performance, such as the rotarod test, and further memory testing, like the Y-maze test, for example, as well as further tests to measure anxiety, like the plus-maze test, could be considered in order to obtain a more complete picture of the effects of long-term fipronil-treatment. In such further studies, behavioral parameters should be measured at more frequent time-points and a longer overall study duration should be considered to validate the gradual increase in symptoms observed in our study. Quantification of fipronil and fipronil-sulfone in brain and adipose tissue should further be considered. In addition, the respective contribution of GABA receptor inhibition and thyroid disruption to the behavioral effects observed following chronic fipronil administration should be characterized more precisely. For this purpose, a supplementary group of fipronil-treated animals receiving concomitant T_4_ replacement could be included in future studies. 

## 5. Conclusions

Our results show that a chronic weekly low-dose treatment with fipronil induces hyperactivity in female mice when tested under moderate stress conditions in the open-field test, which might be caused by a deficit in the capacity to habituate to a novel environment. Fipronil-treatment, however, does not lead to deficits in cognitive function under the conditions applied in our study. The observed effects are most likely caused by the potential accumulation of fipronil sulfone in the brain rather than by the direct effect of the parent compound, which is very rapidly oxidized into fipronil sulfone. 

## Figures and Tables

**Table 1:** Measures of thigmotaxis. Relative time spent (upper value) and distance travelled (lower value) by the animals of the two treatment groups in the different zones of the open-field arena.

Testing Session	Group	Center Zone (%)	Outer Zones (%)
**1** (18 treatments received)	Vehicle (*n* = 10)	20.9 ± 7.4 28.1 ± 4.9	79.1 ± 7.4 71.9 ± 4.9
	Fipronil (*n* = 9)	17.5 ± 10.0 27.2 ± 3.7	81.3 ± 10.2 71.8 ± 4.6
**2** (31 treatments received)	Vehicle (*n* = 10)	11.4 ± 4.8 21.7 ± 6.0	88.6 ± 4.8 78.3 ± 6.0
	Fipronil (*n* = 9)	13.2 ± 5.1 22.7 ± 4.5	86.8 ± 5.1 77.3 ± 4.5
**3** (43 treatments received)	Vehicle (*n* = 10)	12.3 ± 8.3 20.9 ± 8.3	87.7 ± 8.3 79.1 ± 8.3
	Fipronil (*n* = 9)	13.3 ± 3.9 23.2 ± 3.9	86.7 ± 3.9 76.8 ± 3.9

## Appendix A

**Table A1:** Randomization of platform and starting positions in the Morris water maze task during the three sessions of behavior testing.

Visual Platform		Session 1		Session 2		Session 3	
		Platform Position	Starting Position	Platform Position	Starting Position	Platform Position	Starting Position
**Day 0**	**Trial 1**	NW	S	NE	W	NW	NE
	**Trial 2**	SE	NE	E	NW	E	SW
	**Trial 3**	E	N	S	W	S	N
	**Trial 4**	SW	E	N	S	W	SE
	**Trial 5**	N	W	NW	SW	N	E
**Hidden platform**							
**Day 1**	**Trial 1**	W	S	SE	W	NE	S
	**Trial 2**		NE		N		SE
	**Trial 3**		E		SW		W
	**Trial 4**		N		NW		NW
	**Trial 5**		SE		NE		SW
**Day 2**	**Trial 1**		NE		N		SE
	**Trial 2**		E		SW		SW
	**Trial 3**		SE		NE		S
	**Trial 4**		S		W		W
	**Trial 5**		N		NW		NW
**Day 3**	**Trial 1**		N		NW		W
	**Trial 2**		S		W		NW
	**Trial 3**		NE		N		SW
	**Trial 4**		SE		NE		S
	**Trial 5**		E		SW		SE
**Day 4**	**Trial 1**		E		NE		SW
	**Trial 2**		SE		NW		W
	**Trial 3**		N		W		NW
	**Trial 4**		NE		SW		SE
	**Trial 5**		S		N		S
**Day 5**	**Trial 1**		SE		SW		NW
	**Trial 2**		N		NE		S
	**Trial 3**		S		NW		SE
	**Trial 4**		E		N		SW
	**Trial 5**		NE		W		W
**No platform**							
**Day 6**	**Trial 1**	NA	E	NA	NW	NA	SW

NA: not applicable.
